# The NALCN Channel Regulator UNC-80 Functions in a Subset of Interneurons To Regulate *Caenorhabditis elegans* Reversal Behavior

**DOI:** 10.1534/g3.119.400692

**Published:** 2019-11-05

**Authors:** Chuanman Zhou, Jintao Luo, Xiaohui He, Qian Zhou, Yunxia He, Xiaoqin Wang, Long Ma

**Affiliations:** * Center for Medical Genetics, School of Life Sciences,; ‡ Hunan Key Laboratory of Animal Models for Human Diseases,; § Hunan Key Laboratory of Medical Genetics, and; ** Hunan Key Laboratory of Molecular Precision Medicine, Central South University, Changsha, Hunan 410078, China;; † Department of Biomedical Genetics, University of Rochester Medical Center, Rochester, NY 14642

**Keywords:** UNC-80, UNC-79, NCA, NALCN, MeSa avoidance

## Abstract

NALCN (Na^+^ leak channel, non-selective) is a conserved, voltage-insensitive cation channel that regulates resting membrane potential and neuronal excitability. UNC79 and UNC80 are key regulators of the channel function. However, the behavioral effects of the channel complex are not entirely clear and the neurons in which the channel functions remain to be identified. In a forward genetic screen for *C. elegans* mutants with defective avoidance response to the plant hormone methyl salicylate (MeSa), we isolated multiple loss-of-function mutations in *unc-80* and *unc-79*. *C. elegans NALCN* mutants exhibited similarly defective MeSa avoidance. Interestingly, *NALCN*, *unc-80* and *unc-79* mutants all showed wild type-like responses to other attractive or repelling odorants, suggesting that NALCN does not broadly affect odor detection or related forward and reversal behaviors. To understand in which neurons the channel functions, we determined the identities of a subset of *unc-80*-expressing neurons. We found that *unc-79* and *unc-80* are expressed and function in overlapping neurons, which verified previous assumptions. Neuron-specific transgene rescue and knockdown experiments suggest that the command interneurons AVA and AVE and the anterior guidepost neuron AVG can play a sufficient role in mediating *unc-80* regulation of the MeSa avoidance. Though primarily based on genetic analyses, our results further imply that MeSa might activate NALCN by direct or indirect actions. Altogether, we provide an initial look into the key neurons in which the NALCN channel complex functions and identify a novel function of the channel in regulating *C. elegans* reversal behavior through command interneurons.

The NALCN (Na^+^ leak channel, non-selective) channel is a non-selective, voltage-independent cation channel broadly expressed in the animal kingdom (Ren 2011; Liebeskind *et al.* 2012). NALCN functions in neurons to balance the K^+^ leak, set the resting membrane potential, regulate spontaneous firing of neurons and modulate membrane potential in response to environmental stimuli (Ren 2011).

NALCN can affect a variety of biological processes in mammals. NALCN mutant mice die within 24 hr after birth due to disrupted respiratory rhythm (Lu *et al.* 2007). The channel has been implicated in the regulation of pacemaking activity in mouse gastrointestinal tract (Kim *et al.* 2012), clock neuron rhythms (Flourakis *et al.* 2015), firing and glycolytic sensitivity of substantia nigra pars reticulata neurons (Lutas *et al.* 2016), excitability of the retrotrapezoid nucleus neurons (Shi *et al.* 2016), rapid eye movement sleep (Funato *et al.* 2016), and rhythmic stability within the respiratory network (Yeh *et al.* 2017). Mutations in NALCN and its regulatory protein UNC80 are the causes of human diseases (Köroğlu *et al.* 2013; Al-Sayed *et al.* 2013; Perez *et al.* 2016; Stray-Pedersen *et al.* 2016; Fukai *et al.* 2016; Bramswig *et al.* 2018) that are collectively called NALCN channelopathies (Bramswig *et al.* 2018).

NALCN also regulates neuronal activities and behaviors in invertebrates. *Drosophila NALCN* mutants exhibit the narrow abdomen *(na)* phenotype, disrupted circadian rhythm and resistance to the volatile anesthetics halothane (Krishnan and Nash 1990; Nash *et al.* 2002; Lear *et al.* 2005). *C. elegans* has two *NALCN* homologs, *nca-1* and *nca-2*, that function redundantly to regulate the response to volatile anesthetics (Humphrey *et al.* 2007), recycling of synaptic vesicles (Jospin *et al.* 2007), neural circuit activity (Gao *et al.* 2015), motor behavior (Pierce-Shimomura *et al.* 2008), the propagation of neuronal activity from cell bodies to synapses (Yeh *et al.* 2008), ethanol responses (Speca *et al.* 2010) and developmentally timed sleep (Huang *et al.* 2018).

In mammalian cells, NALCN channel can be regulated by G protein-coupled receptor TACR1, Src kinases (Lu *et al.* 2009), a [Ca^2+^]-sensing G protein-coupled receptor (Lu *et al.* 2010) and the M3 muscarinic receptors (M3R) (Swayne *et al.* 2009). In *C. elegans*, NCA channels function downstream of the Gq-Rho pathway (Topalidou *et al.* 2017a), can be negatively regulated by dopamine through the D2-like dopamine receptor DOP-3 (Topalidou *et al.* 2017b) and may interact with the SEK-1 p38 MAPK pathway (Hoyt *et al.* 2017).

Studies in *C. elegans*, *Drosophila* and mice identified the conserved proteins UNC79 and UNC80 (orthologs of *C. elegans* UNC-79 and UNC-80, respectively) as key regulators the NALCN channel (Humphrey *et al.* 2007; Jospin *et al.* 2007; Yeh *et al.* 2008; Pierce-Shimomura *et al.* 2008; Lu *et al.* 2009, 2010; Wang and Ren 2009; Speca *et al.* 2010; Lear *et al.* 2013; Moose *et al.* 2017). In *C. elegans*, loss-of-function (lf) mutants of *unc-79* or *unc-80* phenocopy *nca-2**(lf)*; *nca-1(lf)* double mutants (Humphrey *et al.* 2007; Jospin *et al.* 2007; Yeh *et al.* 2008; Pierce-Shimomura *et al.* 2008; Huang *et al.* 2018). At the molecular level, UNC-79 and UNC-80 are required for the proper expression and axonal localization of NCA channels (Humphrey *et al.* 2007; Jospin *et al.* 2007; Yeh *et al.* 2008). In *Drosophila*, loss-of-function mutations in *unc79*, *unc80* or *NALCN* cause indistinguishable defects in circadian locomotion rhythmicity (Lear *et al.* 2013) and similarly abnormal responses to halothane (Humphrey *et al.* 2007). In mice, UNC79, UNC80 and NALCN form a complex to execute the channel function (Lu *et al.* 2009, 2010). An ER-associated protein, NLF-1, can promote axonal localization of NCA channels in *C. elegans* (Xie *et al.* 2013). The *Drosophila* ortholog of NLF-1 is required for the regulation of circadian neuron excitability (Flourakis *et al.* 2015).

To date, many questions remain to be answered about the function and regulation of the NALCN channel complex, including but not limited to how the channel impacts different behaviors, what neurons mediate the channel functions, how the channel interacts with other neuronal molecules and how the channel is gated. The efficient genetics and well-described neurons of *C. elegans* can be utilized to address some of the questions.

Methyl salicylate (MeSa) is the volatile methyl ester of salicylic acid produced by many plants, and is widely used in medicated oils or mouthwash (Chan 1996; Davis 2007; Lachenmeier *et al.* 2013). In plants, MeSa is required for systemic acquired resistance as a defense to a broad spectrum of pathogens (Park *et al.* 2007, 2009; Tripathi *et al.* 2010; Liu *et al.* 2011b, 2011a). Interestingly, behavioral studies found that MeSa can repel herbivores (Hardie *et al.* 1994; Ulland *et al.* 2008; Snoeren *et al.* 2010) and attract beneficial carnivorous insects (James 2003; De Boer and Dicke 2004; James and Price 2004; Zhu and Park 2005; Lee 2010; Mallinger *et al.* 2011). The molecular and neuronal mechanisms underlying the behavioral effects of MeSa remain unclear.

We previously found that *C. elegans* exhibits a strong avoidance response to MeSa and can be useful for understanding the neuronal effects of MeSa (Luo *et al.* 2015). To identify new genes affecting this behavior, we screened for mutants with defective MeSa avoidance. Surprisingly, the screen isolated novel loss-of-function mutations in *unc-80* and *unc-79*. In this study, we examined how these genes affect *C. elegans* avoidance to MeSa and locomotion. We identified a subset of interneurons that express *unc-80* and analyzed the roles of these neurons in mediating the MeSa avoidance. Our findings suggest that the NALCN complex functions in command interneurons to regulate *C. elegans* reversal behavior.

## Materials and Methods

### Strains

See supplemental Materials and Methods.

### MeSa avoidance assay

*C. elegans* MeSa avoidance assay was performed as previously described (Luo *et al.* 2015). 30 to 200 animals were examined in each experiment.

### Locomotion assay

Synchronized L4 animals were picked into new plates seeded with OP50 bacteria one night before the assay. Body bends were measured by touching an animal on the tail with a worm pick to help initiate locomotion, followed by counting the number of body bends (one head turn) for 1 min.

### Genetic screen for and identification of mutants with defective avoidance response to MeSa

**Figure 1:**
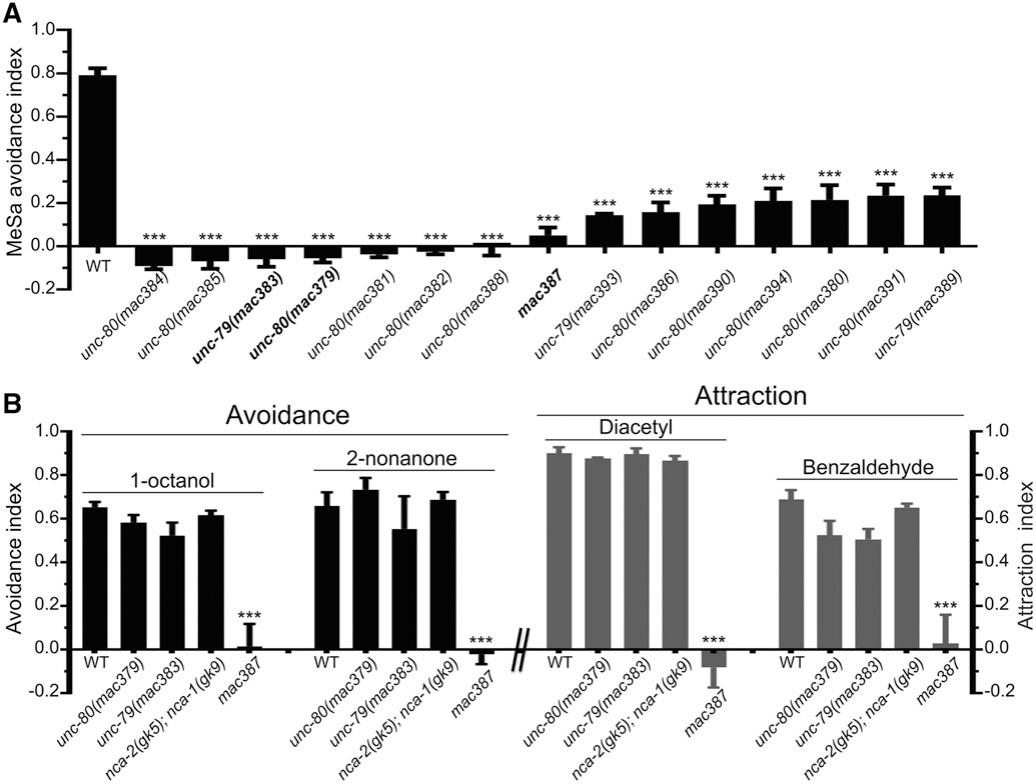
Chemotaxis of mutants with defective MeSa avoidance responses. (A) MeSa avoidance indexes of isolated mutants. (B) Responses of representative mutants to volatile repellents and attractants, including repellents 1-octanol and 2-nonanone and attractants diacetyl and benzaldehyde. Each dataset was based on three biological replicates. Statistics: Bonferroni multiple comparison with one-way ANOVA. ***, *P* < 0.001. Error bars: standard error of mean.

Synchronized L4 wild-type animals (P_0_) were mutagenized with EMS (ethyl methane sulfonate) as described (Brenner 1974). F_1_ progeny were allowed to grow to young adults and bleached to generate synchronized F_2_ progeny for the MeSa avoidance assay. Adult F_2_ animals that failed to avoid MeSa were collected from each individual plate and bleached to generate synchronized adult F_3_ progeny for a new round of MeSa assay. After six rounds of selection, an individual F_7_ progeny that failed to avoid MeSa was picked from each plate, allowed to propagate and retested in the MeSa avoidance assay. From ∼60,000 F_1_ animals, we isolated 15 independent mutants (Figure 1A).

Genetic complementation tests using the response of F_1_ males to MeSa as readout identified three distinct groups, with group one containing 11 mutants (*mac379*, *mac380*, *mac381*, *mac382*, *mac384*, *mac385*, *mac386*, *mac388*, *mac390*, *mac391*, *mac394*), group two containing three mutants (*mac383*, *mac389*, *mac393*) and group three containing one mutant (*mac387*) (Table S1). We selected *mac379*, *mac382* and *mac388* from group one, and *mac383* from group two for genomic sequencing as described (Zhou *et al.* 2018). Sequencing results indicated that *mac379*, *mac382* and *mac388* carried distinct nonsense mutations in the *unc-80* gene and *mac383* carried a nonsense mutation in *unc-79*. The *mac387* mutation in the third complementation group was not further analyzed, as it appeared to affect general chemotaxis of *C. elegans* (Figure 1B).

### Molecular biology

See supplemental Materials and Methods. PCR primers are listed in Table S5.

### Transgene experiments

Germline transgene experiments were performed as described (Mello *et al.* 1991).

For genomic transgene rescue experiments, transgenic mixtures contained 10 to 20 ng/μl genomic PCR fragment and 20 ng/μl pPD95_86 (*Pmyo-3*::*GFP*) as co-injection marker.

For transcriptional reporter experiments, transgene solutions containing 20 to 50 ng/μl reporter construct were injected to wild type.

For neuron-specific knockdown experiments, we used a previously described method with minor modifications (Shen *et al.* 2014). The transgene mixtures contained 20 to 50 ng/μl *Promoter*::*Cas9*::*NLS*::*3′UTR*, 25 ng/μl *PU6*::*unc-79_sgRNA#1* and *#2* each *(or PU6*::*unc-80_sgRNA#1* and *#2* each*)*, and 20 ng/μl pPD95_86 (*Pmyo-3*::*GFP*) or 2.5 ng/μl pCFJ90 (*Pmyo-2*::*mCherry*) as co-injection marker.

For neuron-specific rescue experiments, transgenic mixtures contained 10 to 20 ng/μl *Promoter*::*unc-80_cDNA* and 20 ng/μl pPD95_86 (*Pmyo-3*::*GFP*) or 2.5 ng/μl pCFJ90 (*Pmyo-2*::*mCherry*) as co-injection marker.

### Identification of unc-79- and unc-80-expressing neurons

We used DiI-labeled sensory neurons (Tong and Burglin 2010) as landmarks to facilitate the identification of *P_s_unc-79*-expressing neurons. Images of fluorescence-positive neurons in transgenic animals expressing GFP and/or mCherry reporters were captured using a 63X DIC/fluorescent Leica TCS SP5 II laser confocal microscope and neuronal identities were inferred by overlapping fluorescence signals and by comparing to the anatomical and morphological characteristics of neurons described in Wormatlas (www.wormatlas.org).

### Statistical analysis

*P* values were determined by Paired two-tailed Student’s *t*-test or Bonferroni’s multiple comparison using GraphPad Prism 7.0 software.

### Data availability

Strains and plasmids are available upon request. Supplemental Materials and Methods, supplemental Figures and Tables, and raw data for the behavioral experiments (File S1) and raw images for the neuronal labeling (File S2) can be accessed at figshare: https://doi.org/10.25387/g3.10060007.

## Results

### A screen identified mutants with defective MeSa avoidance response

To identify novel genes affecting the MeSa avoidance behavior, we performed a genetic screen for mutants that failed to avoid MeSa, from which 15 strains were isolated (Figure 1A). Genetic complementation tests divided the mutations to three groups (Table S1).

To investigate whether the mutants had defects in responding to other odorants, we examined a representative mutant in each complementation group (Table S1, *mac379*, *mac383* and *mac387*, respectively) for chemotaxis. *mac379* and *mac383* mutants exhibited wild type-like responses (Figure 1B) to repelling odorants 1-octanol and 2-nonanone, and attractive odorants diacetyl and benzaldehyde (Bargmann *et al.* 1993), suggesting that these mutants have largely normal odorant responses, including odorant detection and odorant-triggered forward or reversal movement. The third mutant, *mac387*, was defective in detecting each odorant (Figure 1B). We postulate that *mac387* might cause broad defects in chemotaxis and did not analyze it further.

### Loss-of-function mutations in unc-80 and unc-79 caused MeSa avoidance defect

To identify the causal mutations leading to the defective MeSa avoidance, we determined the genomic sequences of *mac379*, *mac382* and *mac388* mutants from group 1 and *mac383* from group 2 (Table S1). A comparison of candidate genes found that *unc-80* was the only affected gene shared by *mac379*, *mac382* and *mac388*, with W1524stop, W220stop and W1967stop as the respective mutations (Table S1). Besides W1524stop, *mac379* also carried a missense mutation (G927R) in *unc-80* (Fig. S1A and Table S1). Sanger sequencing identified distinct nonsense or splice site mutations in *unc-80* from other mutants of group 1 (Table S1).

Meanwhile, we identified an R885stop mutation in *unc-79* among the candidate genes for *mac383* (group 2) (Table S1 and Fig. S1B). Considering that *unc-80* and *unc-79* interact with *nca* to affect *C. elegans* behavior (Humphrey *et al.* 2007; Jospin *et al.* 2007; Yeh *et al.* 2008), we speculated that *unc-79* might be the causal gene of *mac383*. Indeed, Sanger sequencing identified distinct deletion/frameshift mutations in *unc-79* from *mac389* and *mac393* mutants of group 2 (Table S1).

**Figure 2:**
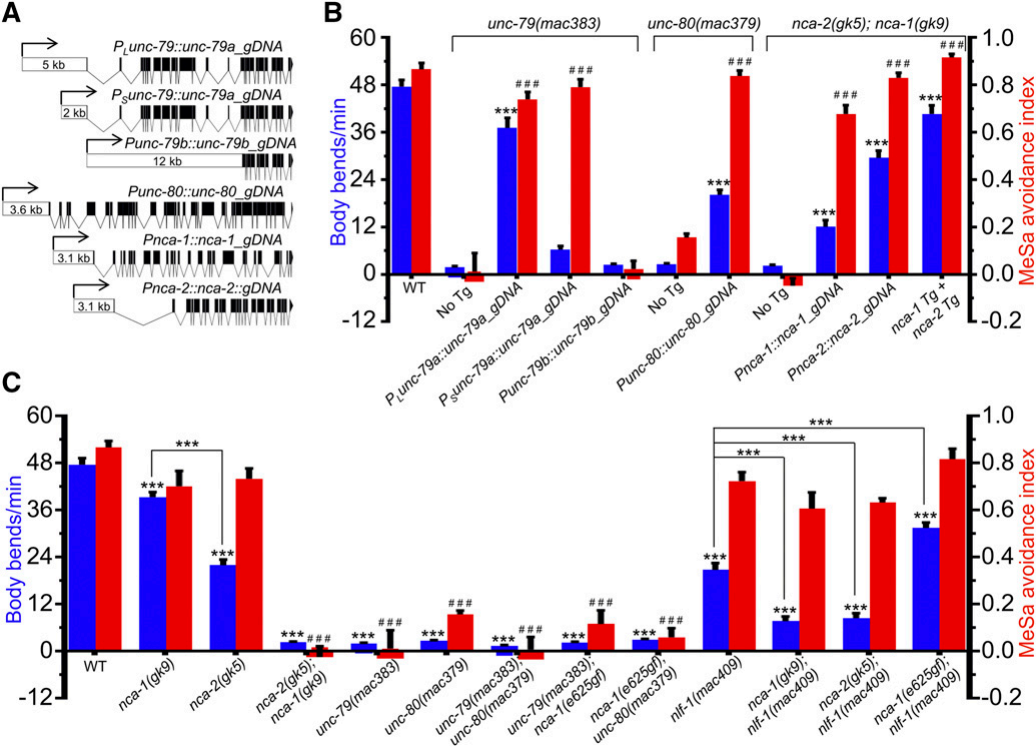
The effects of *unc-79*, *unc-80* and *nca* mutations on MeSa avoidance and locomotion. (A) Transgene structures. Empty boxes indicate promoters. Black boxes indicate exons of each transgene. The length of each promoter is indicated. (B) Transgene rescue of the defective locomotion and the defective MeSa avoidance of *unc-79*, *unc-80* and *nca* loss-of-function mutants. The locomotion (body bends/min, blue columns) and MeSa avoidance indexes (red columns) were based on two independent transgenic lines. For locomotion, 20 animals were assayed for each line with 40 animals assayed in total. For MeSa avoidance indexes, each dataset was based on three biological replicates for each line with six in total. Statistics: Student’s *t*-test and Bonferroni multiple comparison with one-way ANOVA. *** or ^###^, *P* < 0.001. Error bars: standard error of mean. Comparisons were made between transgenic lines and their respective loss-of-function mutants. (C) The locomotion (body bends/min, blue columns, 40 animals per strain) and MeSa avoidance indexes (red columns, three to six biological replicates) of single and double mutants. Genotypes are labeled at bottom. Statistics: Bonferroni multiple comparison with one-way ANOVA. *** or ^###^, *P* < 0.001. Error bars: standard error of mean. Note: data for wildtype, *unc-79**(lf)*, *unc-80**(lf)* and *nca(lf)* animals were repeatedly used for comparison purpose.

To verify that *unc-79* is required for the MeSa avoidance behavior, we performed transgene rescue experiments. Driven by a long endogenous promoter (Figure 2A, *P_L_unc-79a*, 5.0 kb), an *unc-79a* gDNA transgene (Figure 2A) strongly rescued the defective locomotion and MeSa avoidance of *unc-79**(**mac383**)* mutants (Figure 2B). Driven by a short promoter (Figure 2A, *P_S_unc-79a*, 2.0 kb), the *unc-79a* gDNA transgene only recued the defective MeSa avoidance but not the defective locomotion (Figure 2B). We also examined a transgene that covers the shorter *unc-79b* isoforms (Fig. S1B and 2A, *Punc-79b*) and found that it failed to rescue either defective behavior (Figure 2B).

Similarly, an *unc-80* gDNA transgene that covers all annotated *unc-80* isoforms (Fig. S1A) driven by an *unc-80* endogenous promoter (Figure 2A, *Punc-80*, 3.6 kb) can significantly rescue the defective locomotion and MeSa avoidance of *unc-80**(**mac379**)* mutants (Figure 2B). Compared to the fully rescued MeSa avoidance, the defective locomotion was only partially rescued (Figure 2B).

*unc-79* and *unc-80* loss-of-function mutants are hypersensitive to the anesthetic halothane and exhibit the “fainter” phenotype (Morgan and Cascorbi 1985; Sedensky and Meneely 1987; Morgan *et al.* 1988, 2007; Humphrey *et al.* 2007; Jospin *et al.* 2007; Yeh *et al.* 2008). Similar “fainter” phenotype was observed in all *unc-80* and *unc-79* mutants isolated in this study. In addition, *unc-79**(**e1291**)* and *unc-80**(**e1272**)* animals, two previously described loss-of-function mutants (Jospin *et al.* 2007), exhibited defective MeSa avoidance resembling that of *unc-79**(**mac383**)* or *unc-80**(**mac379**)* mutants (File S1, additional raw data). Together, these results suggest that *unc-79* and *unc-80* are specifically required for *C. elegans* MeSa avoidance behavior and we isolated loss-of-function mutations in *unc-79* and *unc-80*.

### nca-1 and nca-2 were redundantly required for the MeSa avoidance behavior

*C. elegans nca-1* and *nca-2* encode functionally redundant NALCN channels (Humphrey *et al.* 2007; Morgan *et al.* 2007; Jospin *et al.* 2007; Yeh *et al.* 2008). To investigate whether *nca* is required for the MeSa avoidance behavior, we examined *nca* single and double mutants. We found that either *nca-1(**gk9lf**)* or *nca-2**(**gk5lf**)* single mutants exhibited wild type-like MeSa avoidance (Figure 2C), while *nca-2**(lf)*; *nca-1(lf)* double mutants were strongly defective in avoiding MeSa (Figure 2B and 2C). An *nca-1* gDNA transgene (Figure 2A, *Pnca-1*), an *nca-2* gDNA transgene (Figure 2A, *Pnca-2*), or both transgenes together can strongly rescue the defective MeSa avoidance of the double mutants (Figure 2B). Though the locomotion defect of the double mutants was significantly rescued by either transgene (body bends/min, see Materials and Methods), the two transgenes together appeared to have a stronger effect (Figure 2B). Similar to *unc-79**(lf)* or *unc-80**(lf)* mutants, *nca(lf)* double mutants exhibited normal responses to other attractants and repellents (Figure 1B).

A gain-of-function (gf) mutation in *nca-1*, *e625*, causes the “coiler” phenotype (Yeh *et al.* 2008). To understand how *unc-79* or *unc-80* interacts with *nca-1(**e625gf**)* in affecting the MeSa avoidance, we generated double mutants. Consistent with previous findings (Yeh *et al.* 2008), we found that *unc-79**(lf)* or *unc-80**(lf)* completely suppressed the “coiler” phenotype of the *nca-1(gf)* mutants. The double mutants also exhibited defective MeSa avoidance (Figure 2C).

The ER protein NCA localization factor-1 (NLF-1) is required for axonal localization of NCA proteins (Xie *et al.* 2013). To examine whether *nlf-1* is required for the MeSa avoidance, we generated three *nlf-1* deletion lines (Table S2) using a CRISPR/Cas9 method (Friedland *et al.* 2013). Taking *nlf-1**(**mac409**)* as the representative loss-of-function allele (Figure 2C), we found that *nlf-1**(**mac409lf**)* itself, or together with *nca-1(lf)*, *nca-2**(lf)* or *nca-1(gf)*, did not cause obviously defective MeSa avoidance (Figure 2C). We observed that *nlf-1**(lf)* can suppress the “coiler” phenotype of *nca-1(gf)* mutants, consistent with previous findings (Xie *et al.* 2013).

To understand how these genes affect other behaviors, we examined the locomotion of the mutants. *unc-79**(lf)* and *unc-80**(lf)* single mutants, or double mutants carrying either *unc-79**(lf)* or *unc-80**(lf)*, all exhibited severely defective locomotion (Figure 2C).

Interestingly, *nca-1(lf)* single mutants exhibited weakly defective locomotion, while *nca-2**(lf)* single mutants were moderately defective (Figure 2C). *nca(lf)* double mutants had severely defective locomotion similar to that of *unc-79**(lf)* or *unc-80**(lf)* mutants (Figure 2C). We found that *nlf-1**(lf)* mutants exhibited a moderately defective locomotion, which can be enhanced by *nca-1(lf)* or *nca-2**(lf)* (Figure 2C). However, the defective locomotion of *nlf-1**(lf)* mutants appeared to be partially improved by *nca-1(gf)* (Figure 2C).

### The identification of a subset of unc-80-expressing neurons

Previous studies found that *unc-80*, *unc-79* and *nca* are broadly expressed in *C. elegans* neurons (Jospin *et al.* 2007; Yeh *et al.* 2008). However, the classes of most individual neurons remain to be identified. To understand the neuronal mechanism underlying the function of the NCA channel complex, we generated transgenic animals expressing GFP driven by *Punc-80* (Figure 2A). In these animals, GFP was observed in multiple neurons, ventral nerve cord and vulval muscles (Fig. S2A), consistent with previous findings (Jospin *et al.* 2007; Yeh *et al.* 2008).

**Figure 3:**
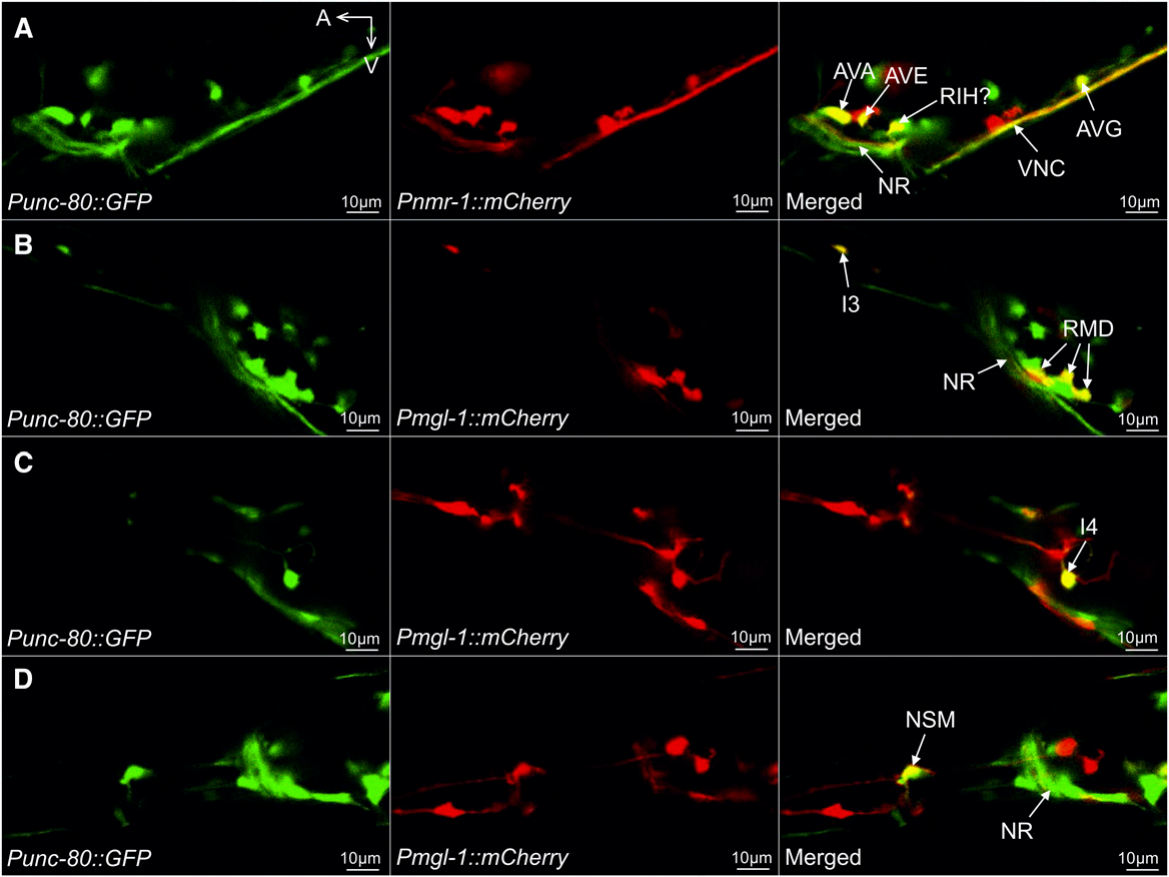
Neuronal double-labeling identified a subset of *unc-80*-expressing neurons. (A) GFP expression driven by the *unc-80* promoter (left panel, *Punc-80*), mCherry driven by the *nmr-1* promoter (middle panel, *Pnmr-1*) and the merged image (right panel) showing GFP expression in AVA, AVE and AVG neurons and probably in the RIH neuron. (B, C, D) GFP driven by *Punc-80* (left panels), mCherry driven by *Pmgl-1* (middle panels) and the merged images showing GFP expression in RMD and I3 (B, right panel), I4 (C, right panel) and NSM (D, right panel) neurons. VNC: ventral nerve cord. NR: nerve ring. A: anterior. V: ventral. Results were based on three independent transgenic lines. Pictures were taken from a line with the most robust expression of reporters.

We next used transgene double-labeling to identify individual neurons that express *unc-80*. In animals co-expressing *Punc-80*::*GFP* (Figure 3A, left panel) and *Pnmr-1*::*Cherry* (Figure 3A, middle panel) (neurons labeled by *Pnmr-1* are listed in Table S3) (Brockie *et al.* 2001b), we observed visible GFP expression in AVA, AVE and AVG neurons (Figure 3A, right panel) but no obvious expression in AVD and RIM neurons, two classes of neurons also reported to be labeled by *Pnmr-1*. In addition, a neuron similar to RIH appeared to be consistently labeled by GFP and mCherry (Figure 3A, right panel). Additional double-labeling using *Punc-80*::*GFP* (Figure 3B, 3C and 3D, left panels) with mCherry driven by the *mgl-1* promoter (Table S3) (Wenick and Hobert 2004; Greer *et al.* 2008) (Figure 3B, 3C and 3D, middle panels) identified four other classes of GFP-expressing neurons, including RMD and I3 (Figure 3B, right panel), I4 (Figure 3C, right panel) and NSM (Figure 3D, right panel). Except for these neurons, multiple other *GFP*-expressing neurons remain to be identified.

Using a previously described *nlf-1* promoter (Xie *et al.* 2013) to drive the *mCherry* transgene, we confirmed the expression of *nlf-1* in head neurons, ventral nerve cord and tail neurons (Fig. S2B) (Xie *et al.* 2013). Neuronal double-labeling found that AVA and AVE neurons, among other unidentified neurons, were co-labeled by *Punc-80*::*GFP* and *Pnlf-1*::*mCherry* (Fig. S3A). The expression of *nlf-1* in AVA and AVE was also described previously (Xie *et al.* 2013).

### Neuron-specific transgene rescued the defective MeSa avoidance of unc-80(lf) mutants

**Figure 4:**
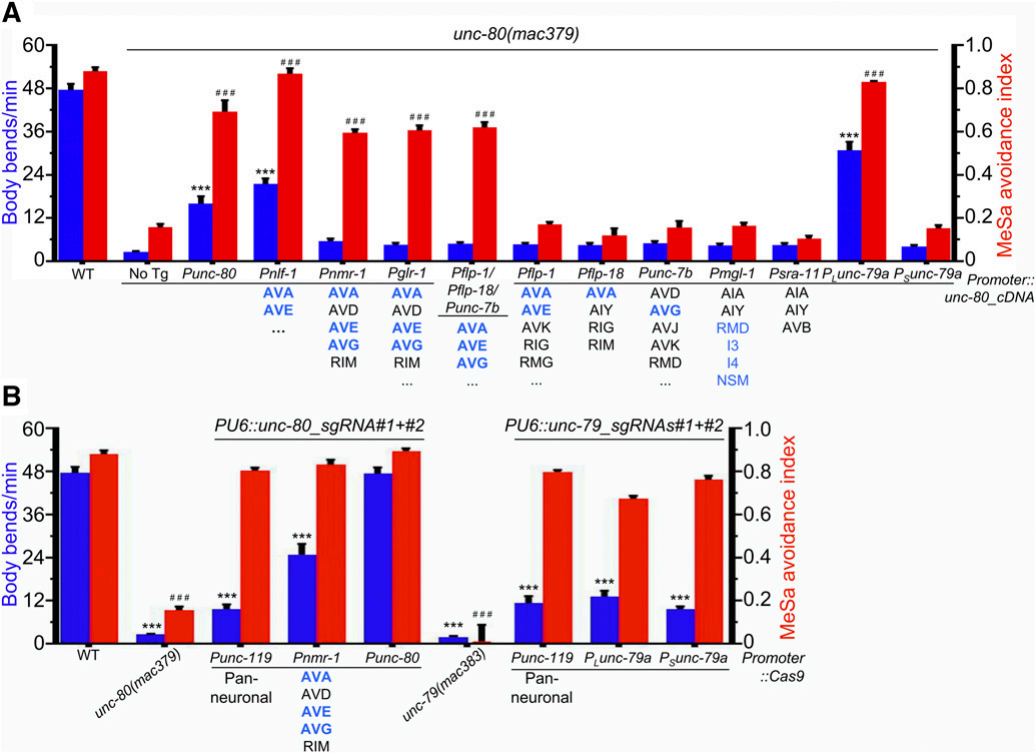
Neuron-specific transgene rescue and neuron-specific knockdown experiments. (A) The locomotion (body bends/min, blue columns, 20 animals per transgenic line, 40 animals in total) and MeSa avoidance indexes (red columns) of *unc-80**(lf)* transgenic lines expressing an *unc-80* cDNA transgene driven by different promoters compared to *unc-80**(lf)* mutants. *unc-80*-expressing neurons are highlighted in blue. Promoter types and the corresponding neurons are labeled at bottom. Results were based on two independent transgenic lines. (B) The locomotion (body bends/min, blue columns, 20 animals per transgenic line, 40 animals in total) and MeSa avoidance indexes (red columns) of transgenic knockdown lines expressing *Cas9* driven by different promoters and *unc-80*-targeting sgRNAs or *unc-79*-targeting sgRNAs. Results were based on two independent lines for each transgene. Comparisons were made with wild type. Statistics: Bonferroni multiple comparison with one-way ANOVA. *** or ^###^, *P* < 0.001. Error bars: standard error of mean. Note: data for wildtype, *unc-79**(lf)* and *unc-80**(lf)* animals were repeatedly used for comparison purpose.

To understand the function of *unc-80* in different neurons, we performed neuron-specific transgene rescue experiments. An *unc-80* cDNA transgene driven by *Punc-80* can significantly rescue the defective locomotion and MeSa avoidance of *unc-80**(lf)* mutants (Figure 4A, *Punc-80*). Still, the locomotion was partially rescued while the MeSa avoidance was more strongly rescued. The *nlf-1* promoter had a similar rescuing effect as the *unc-80* promoter (Figure 4A, *Pnlf-1*), consistent with the result that *unc-80* and *nlf-1* were co-expressed in multiple neurons (Fig. S3A).

To examine whether the *unc-80* transgene might be effective in a subset of *unc-80*-expressing neurons, we tested the *nmr-1* promoter (Table S3). The *Pnmr-1*::*unc-80* transgene significantly rescued the defective MeSa avoidance but not the defective locomotion of *unc-80**(lf)* mutants (Figure 4A, *Pnmr-1*). The *glr-1* promoter (Table S3), which also drives transgene expression in AVA, AVE and AVG neurons (Brockie *et al.* 2001a), similarly rescued the defective MeSa avoidance but not the defective locomotion (Figure 4A, *Pglr-1*).

To determine whether the *unc-80* transgene can be effective in a smaller set of neurons, we tested three other promoters, *flp-1*, *flp-18* and *unc-7b* (Table S3) (Nelson *et al.* 1998; Rogers *et al.* 2003; Altun *et al.* 2009), which label *unc-80*-expressing AVA/AVE, AVA, and AVG neurons, respectively. Each transgene by itself failed to rescue either defective behavior (Figure 4A, *Pflp-1*, *Pflp-18* or *Punc-7b*). However, when injected together, the three transgenes can significantly rescue the defective MeSa avoidance of *unc-80**(lf)* mutants (Figure 4A, *Pflp-1/Pflp-18/Punc-7b*). Similar to the *nmr-1* or the *glr-1* promoter, the defective locomotion was not rescued (Figure 4A, *Pflp-1/Pflp-18/Punc-7b*).

To understand whether other neurons might be involved in the MeSa avoidance behavior, we tested the *mgl-1* promoter, which drives overlapping expression with *Punc-80* in RMD, I3, I4 and NSM neurons (Figure 3B, 3C and 3D). However, the *Pmgl-1*::*unc-80* transgene failed to rescue either the defective MeSa avoidance or the defective locomotion of *unc-80**(lf)* mutants (Figure 4A, *Pmgl-1*). The *sra-11* promoter (Table S3) (Altun-Gultekin *et al.* 2001) did not rescue the defective behaviors either (Figure 4A, *Psra-11*), consistent with our finding that its expression did not obviously overlap that of *Punc-80* (Fig. S3B).

### Neuron-specific knockdown of unc-80 caused defective locomotion

To investigate whether *unc-80* expression in specific neurons is essential for its function, we used a CRISPR/Cas9-based method (Shen *et al.* 2014) to examine whether neuron-specific knockdown of *unc-80* would phenocopy the behavioral defects of *unc-80**(lf)* mutants.

Transgenic animals expressing two *unc-80*-targeting sgRNAs (Table S4) and *Cas9* driven by the pan-neuronal *unc-119* promoter (Table S3) (Maduro and Pilgrim 1995) exhibited obviously defective locomotion (Figure 4B, *Punc-119*). Limiting the expression of *Cas9* to a subset of interneurons, *e.g.*, AVA, AVE and AVG, using the *nmr-1* promoter also caused defective locomotion (Figure 4B, *Pnmr-1*). However, *Punc-80*::*Cas9* failed to cause obviously defective locomotion (Figure 4B, *Punc-80*). We postulate that the expression of *Punc-80*::*Cas9* might not be sufficient in disrupting the *unc-80* locus in these animals.

Interestingly, different from the locomotion, the MeSa avoidance response was not affected in any of the knockdown lines (Figure 4B, red columns).

### unc-79 and unc-80 functioned in overlapping neurons

Though it is widely assumed that UNC-79 and UNC-80 function together in the same neurons, there is limited genetic evidence supporting this notion. To validate this assumption, we generated transgenic lines expressing GFP driven by the *P_L_unc-79a* promoter (Figure 2A). In these animals, GFP was expressed in multiple head and tail neurons, ventral nerve cord and vulval muscles (Fig. S2C), a pattern similar to that of *unc-80* (Fig. S2A). Using *P_L_unc-79a* to drive an *unc-79a_cDNA*::*GFP* transgene (Fig. S4A), we found similar expression of the UNC-79a::GFP fusion protein in head neurons (Fig. S4B), ventral nerve cord (Fig. S4B), vulval muscles (Fig. S4C), motor neurons (Fig. S4C) and tail neurons (Fig. S4D). This transgene also significantly rescued the defective MeSa avoidance and locomotion of *unc-79**(lf)* mutants (File S1, additional raw data), similar to that of the *unc-79a_gDNA* transgene (Figure 2B).

We next examined the expression of the *P_S_unc-79a* promoter. Interestingly, GFP driven by *P_S_unc-79a* was only detected in several pairs of head neurons (Fig. S2D), a pattern similar to that described by Humphrey *et al.* (Humphrey *et al.* 2007). The expression of *P_S_unc-79a*::*GFP* in anterior and posterior portions of the intestine (Fig. S2D) was likely non-specific as *P_L_unc-79a*::*GFP* was not detected there (Fig. S2C).

**Figure 5:**
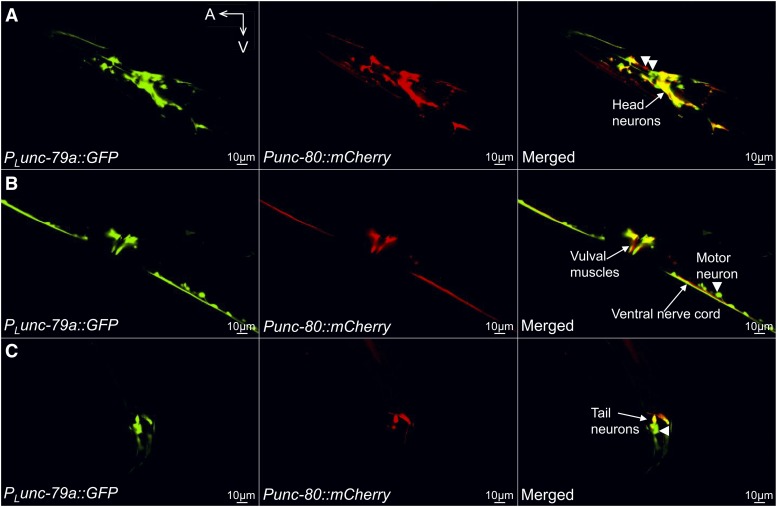
Neuronal double-labeling found that *unc-79* and *unc-80* were expressed in largely overlapping neurons. (A) GFP expression driven by the long *unc-79a* promoter (left panel, *P_L_unc-79a*), mCherry driven by the *unc-80* promoter (middle panel, *Punc-80*) and the merged image (right panel) showing multiple head neurons co-expressing GFP and mCherry. (B) Same as (A), showing vulval muscles co-labeled by GFP and mCherry. (C) Same as (A, B), showing a few tail neurons co-labeled by GFP and mCherry. Arrow heads point to neurons that appear to express only one fluorescent reporter. A: anterior. V: ventral. Results were based on three independent transgenic lines. Pictures were taken from a line with the most robust expression of reporters.

To investigate whether *unc-79* and *unc-80* were expressed in the same neurons, we generated transgenic animals co-expressing *P_L_unc-79a*::*GFP* and *Punc-80*::*mCherry*. In these animals, GFP and mCherry co-labeled a large number of head neurons (Figure 5A), the vulval muscles (Figure 5B) and a few tail neurons (Figure 5C). One or more head neurons (Figure 5A, right panel, arrow heads), a motor neuron (Figure 5B, right panel, arrow head) and a tail neuron (Figure 5C, right panel, arrow head) appeared to be labeled by only GFP or mCherry but not both, which might be caused by mosaicism or variable expression of the transgenes. A *P_L_unc-79a*::*unc-80* cDNA transgene could significantly rescue the defective locomotion and MeSa avoidance of *unc-80**(lf)* mutants (Figure 4A, *P_L_unc-79a*), consistent with the overlapping expression of the two genes.

The limited expression pattern of the *P_S_unc-79a* promoter (Fig. S2D) and its partial rescuing strength (Figure 2B) suggest that it might define a subset of functioning *unc-79* neurons. We combined DiI tracing and neuronal double-labeling to identify the neurons labeled by this promoter.

**Figure 6:**
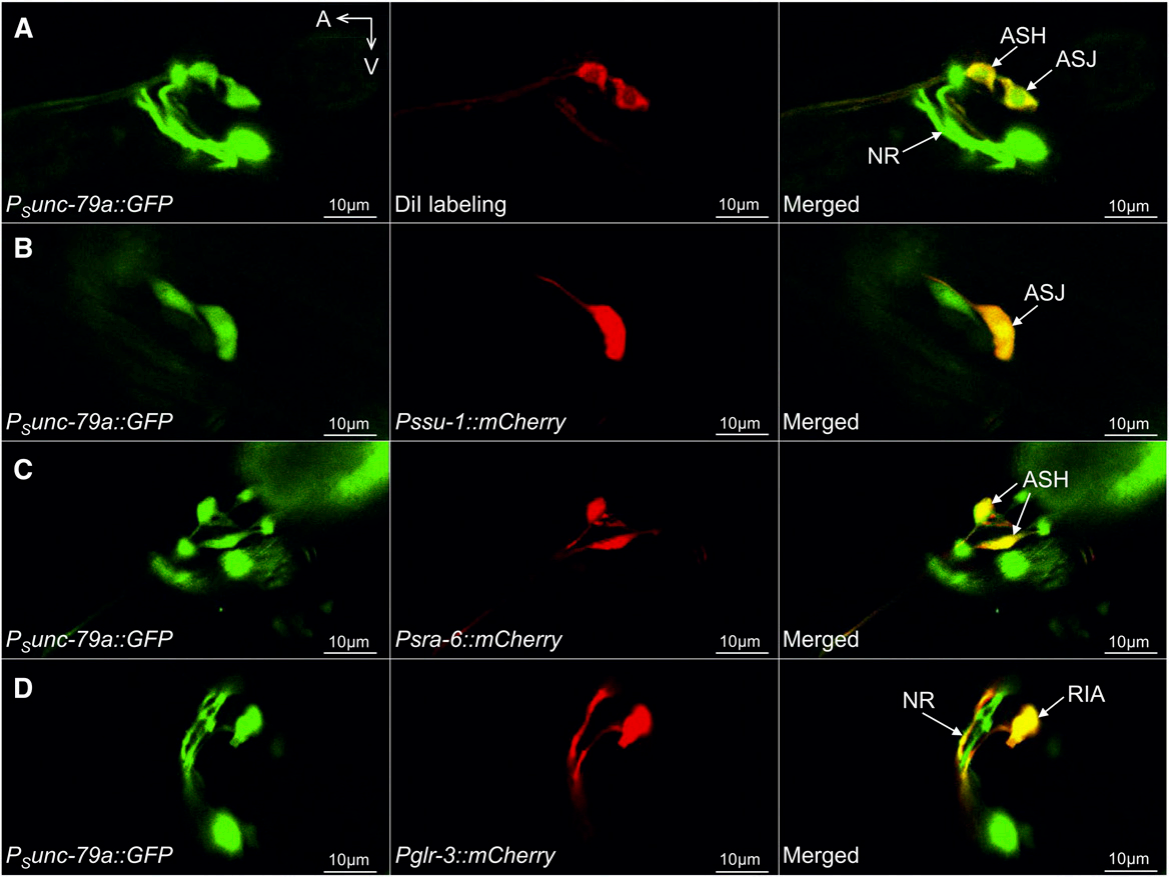
DiI tracing and neuronal double-labeling identified neurons labeled by the short *unc-79a* promoter. (A) GFP expression driven by the short *unc-79a* promoter (left panel, *P_S_unc-79a*), DiI labeling of sensory neurons (middle panel) and the merged image (right panel) showing GFP expression in ASJ and ASH neurons. (B, C, D) GFP driven by *P_S_unc-79a* (B, C, D, left panels), mCherry driven by *Pssu-1* (B, middle panel), *Psra-6* (C, middle panel) or *Pglr-3* (D, middle panel) and the merged images showing GFP expression in ASJ neurons (B, right panel), ASH neurons (C, right panel) and RIA neurons (D, right panel). NR: nerve ring. A: anterior. V: ventral. Results were based on three independent transgenic lines. Pictures were taken from a line with the most robust expression of reporters.

In *P_S_unc-79a*::*GFP* transgenic animals, we found that GFP co-labeled ASH and ASJ that were also stained with DiI (Figure 6A). The expression of *P_S_unc-79a*::*GFP* (Figure 6B and 6C, left panels) in ASJ (Figure 6B, right panel) and ASH (Figure 6C, right panel) was verified by co-labeling with *Pssu-1*::*mCherry* (Table S3) (Carroll *et al.* 2006) (Figure 6B, middle panel) and *Psra-6*::*mCherry* (Table S3) (Troemel *et al.* 1995) (Figure 6C, middle panel), respectively. *P_S_unc-79a*::*GFP* (Figure 6D, left panel) also appeared to label the RIA neurons (Figure 6D, right panel), which were identified by co-labeling with *Pglr-3*::*mCherry* (Table S3) (Brockie *et al.* 2001a) (Figure 6D, middle panel). The fourth pair of neurons labeled by *P_S_unc-79a*::*GFP* was similar to either of the closely positioned motor neurons RMF or RMH (Fig. S3C). Unlike the *P_L_unc-79a* promoter, a *P_S_unc-79a*::*unc-80* transgene failed to rescue the defective locomotion or MeSa avoidance of *unc-80**(lf)* mutants (Figure 4A, *P_s_unc-79a*), consistent with the finding that *P_S_unc-79a*::*mCherry* and *Punc-80*::*GFP* did not appear to co-label neurons (Fig. S3D).

We next used the CRISPR/Cas9 knockdown to investigate the function of *unc-79* in neurons. Transgenic animals expressing two *unc-79*-targeting sgRNAs (Table S4) and the pan neuronally expressed *Punc-119*::*Cas9* exhibited significantly defective locomotion (Figure 4B, *Punc-119*). *Cas9* driven by the long or short *unc-79* promoter also caused defective locomotion (Figure 4B, *P_L_unc-79a and P_S_unc-79a*). However, none of the *unc-79*-knockdown lines exhibited obviously defective MeSa avoidance (Figure 4B, red columns), which is similar to *unc-80*-knockdown animals.

## Discussion

In this study, we found that *unc-79*, *unc-80* and the *nca* genes are specifically required for *C. elegans* avoidance response to methyl salicylate. We verified that *unc-79* and *unc-80* are expressed and function in overlapping neurons. We identified and examined the functional relevance of a subset of *unc-80*-expressing neurons. Our findings suggest a novel role of the *NALCN*-related genes in the command interneurons AVA and AVE to regulate *C. elegans* reversal behavior.

Genetic, cellular, biochemical and electrophysiological studies suggest that UNC79, UNC80 and NALCN form a channel complex to regulate resting membrane potential and neuronal excitability (Humphrey *et al.* 2007; Lu *et al.* 2007, 2009, 2010; Jospin *et al.* 2007; Yeh *et al.* 2008; Wang and Ren 2009; Lear *et al.* 2013; Xie *et al.* 2013). Mutations of these genes in *C. elegans* and *Drosophila* often cause closely similar phenotypes (Humphrey *et al.* 2007; Jospin *et al.* 2007; Yeh *et al.* 2008; Lear *et al.* 2013; Xie *et al.* 2013; Moose *et al.* 2017), exemplifying the functional interdependency of these proteins. In human, UNC80 and NALCN mutations are the causes of complex syndromic diseases (Köroğlu *et al.* 2013; Al-Sayed *et al.* 2013; Perez *et al.* 2016; Stray-Pedersen *et al.* 2016; Fukai *et al.* 2016; Bramswig *et al.* 2018) that are collectively called NALCN channelopathies (Bramswig *et al.* 2018). A notion derived from these studies is that NALCN, UNC80 and UNC79 should be expressed in the same neurons to perform their functions. However, a closer look at their co-expression in neurons has been insufficient. Here, we provide both neuronal identification and transgene rescue results to verify the co-expression and function of *unc-80* and *unc-79* in the same neurons. Though not surprising, our work could be useful for analyzing the neuronal functions of these genes in *C. elegans*.

*unc-79* and *unc-80* loss-of-function mutants exhibited indistinguishable MeSa-specific avoidance defects, a phenotype that is also shared by the *NALCN* mutants (*nca*). Interestingly, all these mutants displayed wild type-like responses to other attractants or repellents, suggesting they had largely normal odorant sensation and forward/reversal behaviors. Therefore, MeSa might trigger an avoidance response that is specifically mediated by the NALCN channel complex.

Our genetic and neuronal analyses suggest that *unc-80* expression in a subset of *unc-80*-expressing neurons, *e.g.*, in the command interneurons AVA and AVE and the anterior guidepost neuron AVG defined by the *nmr-1* or *glr-1* promoter, is sufficient for eliciting the MeSa avoidance. However, unlike the endogenous *unc-79*, *unc-80* or *nlf-1* promoters, the *nmr-1* or *glr-1* promoter did not rescue the defective locomotion. This result is consistent with the study of *nlf-1*, in which Xie *et al.* (Xie *et al.* 2013) found that an *Pnmr-1*::*nlf-1* transgene alone was not sufficient in rescuing the defective body bending frequency of *nlf-1**(lf)* mutants, while a combination of *Pnmr-1*::*nlf-1* and *Psra-11*::*nlf-1* transgenes would do. Therefore, broader expression of *unc-80* in more interneurons might be necessary for generating a normal locomotion.

The key role of AVA in mediating NALCN functions was also suggested by two recent studies, in which the authors found that *nlf-1* was expressed in AVA and AVE to regulate *C. elegans* locomotion (Xie *et al.* 2013) and NCA can activate the AVA neurons to potentiate persistent motor circuit activity in *C. elegans* (Gao *et al.* 2015). Together, our findings suggest a neural network containing at least AVA, AVE and AVG in regulating *C. elegans* behaviors.

AVA neurons are key regulators of a variety of *C. elegans* behaviors, *e.g.*, touch-induced movement (Chalfie *et al.* 1985; White *et al.* 1986), reversal locomotion (Pokala *et al.* 2014), mechanosensory habituation (Sugi *et al.* 2014), variability in reversal response to odor stimuli (Gordus *et al.* 2015) and repetitive reversals caused by glutamate spillover (Katz *et al.* 2019). The expression of *unc-80* and presumably *unc-79* and *nca* in AVA neurons implicate the NALCN channel complex as a regulator of these behaviors.

MeSa was first isolated in the 19^th^ century and has been used as a natural flavoring agent and topical pain relief for decades (https://pubchem.ncbi.nlm.nih.gov/compound/methyl_salicylate). Recent studies suggest that MeSa is synthesized by many plants to signal the systemic acquired resistance to multiple pathogens (Liu *et al.* 2011a). Surprisingly, MeSa can attract beneficial carnivorous insects and also repel herbivores (Hardie *et al.* 1994; James 2003; De Boer and Dicke 2004; James and Price 2004; Zhu and Park 2005; Ulland *et al.* 2008; Lee 2010; Snoeren *et al.* 2010; Mallinger *et al.* 2011). We were particularly intrigued by the latter findings and established the *C. elegans* MeSa avoidance assay to study the genetics underlying the behavioral effects of MeSa (Luo *et al.* 2015). In our previous study, neuron-specific rescue experiments suggest that the *npr-1*-expressing inter/motor neurons RMG and the *npr-2*-expressing interneurons AIZ might be involved in the MeSa avoidance behavior (Luo *et al.* 2015). Genetic mutants lacking sensory neurons imply that AWB might play a major role in detecting MeSa, while AWC might play a minor role (Luo *et al.* 2015). Together, the involvement of AWC, AIZ and AVA neurons in mediating the MeSa avoidance is consistent with the placement of these neurons in a core circuit that regulates *C. elegans* chemotaxis (Gordus *et al.* 2015).

Previously, the human TRPV1 channel was shown to be inhibited by MeSa (Ohta *et al.* 2009). However, the wild type-like response to MeSa by two TRPV channel mutants (*osm-9* and *ocr-2*) (Luo *et al.* 2015) and the finding that all five TRPV channel expression was not detected in AWB neurons (Colbert *et al.* 1997; Tobin *et al.* 2002) do not support a role of a TRPV channel as the MeSa receptor in *C. elegans*. Alternatively, a GPCR expressed in antenna sensory neurons of the tortricid moth was found to exhibit high sensitivity to MeSa in the insect sf9 cells (Jordan *et al.* 2009). This result is consistent with the findings that GPCRs can regulate NALCN activities in mammals (Lu *et al.* 2009, 2010; Swayne *et al.* 2009; Yeh *et al.* 2017; Philippart and Khaliq 2018), NCA are downstream targets of neuronal G-protein signals in *C. elegans* (Topalidou *et al.* 2017a, 2017b) and GPCR signals are involved in *C. elegans* avoidance response to MeSa (Luo *et al.* 2015). Therefore, a GPCR might act as the MeSa receptor in certain *C. elegans* sensory neurons.

We made a few intriguing findings in this study. First, restoring *unc-80* expression specifically in interneurons using *Pnmr-1*, *Pglr-1* or *Pflp-1/Pflp-18/Punc-7b* significantly rescued the defective MeSa avoidance of *unc-80**(lf)* mutants, suggesting that NALCN expression in interneurons shared by these promoters but not in other neurons might be sufficient for MeSa to elicit the reversal behavior. Second, *unc-79* transgene expression driven by the *P_s_unc-79a* promoter could strongly rescue the defective MeSa avoidance of *unc-79**(lf)* mutants, suggesting that a limited restoration of NALCN function, potentially only in sensory neurons ASH and ASJ and two pairs of interneurons (RIA and RMF/RMH) but not in other classes of neurons, might be sufficient for MeSa to trigger the reversal behavior. Third, only the locomotion but not the MeSa avoidance behavior was significantly affected by neuron-specific knockdown of *unc-80* or *unc-79*, suggesting that partial expression of the genes due to incomplete knockdown is sufficient for MeSa to elicit a strong avoidance response.

There results prompt us to postulate that MeSa might cause the avoidance behavior by acting on more than one component, *e.g.*, a group of neurons, of a multi-component neural network controlling the reversal behavior. In this network, the expression of the NALCN complex in any MeSa-responding components would be sufficient for triggering the avoidance behavior. Only when the NALCN complex is absent in all MeSa-responding components, the animals would exhibit a defective avoidance response. In addition, a remote possibility derived from these findings is that MeSa might act on the NALCN complex as an agonist. Therefore, a continuing investigation on the molecular mechanism underlying the MeSa avoidance behavior is warranted for further understanding of the function of the NALCN complex.

Finally, that MeSa might activate the NALCN channel complex provides a potential molecular explanation on why MeSa can repel herbivores and attract beneficial insects. For example, herbivores might express NALCN on neurons that promote avoidance behavior, while beneficial insects might express NALCN on neurons that promote attraction behavior.

## Conclusion

In short, we found that *unc-79*, *unc-80* and *nca* genes are specifically required for *C. elegans* avoidance response to the plant hormone MeSa. We verified that *unc-79* and *unc-80* are co-expressed and function in overlapping neurons. The command interneurons AVA, AVE and the guidepost neuron AVG can be sufficient for *unc-80* regulation of the MeSa avoidance. Our results suggest a novel function of the NALCN complex expressed in command interneurons as a regulator of *C. elegans* reversal behavior.
